# Searching by grant number: comparison of funding acknowledgments in NIH RePORTER, PubMed, and Web of Science

**DOI:** 10.5195/jmla.2019.554

**Published:** 2019-04-01

**Authors:** Kimberly Powell

**Affiliations:** Research Impact Informationist, Woodruff Health Sciences Center Library, Emory University, Atlanta, GA krpowel@emory.edu

## Abstract

**Objective:**

Several publication databases now index the associated funding agency and grant number metadata with their publication records. Librarians who are familiar with the particulars of these databases can assist investigators and administrators with data gathering for publication summaries and metrics required for renewals of and progress reports for National Institutes of Health (NIH) grants.

**Methods:**

Publication lists were pulled from three main indexers of publication-associated funding information (NIH RePORTER, PubMed, and Web of Science), using iterative search strategies. All discovered variations for the cited grant number of interest were recorded and tested. Publication lists were compared for overall coverage.

**Results:**

A total of 986 publications citing the single grant number of interest were returned from the given time frame: 920 were found in PubMed, 860 in NIH RePORTER, and 787 in Web of Science. Web of Science offered the highest percentage of publications that were not found in the other 2 sources (n=63). Analysis of publication funding acknowledgments uncovered 21 variations of the specific NIH award of interest that were used to report funding support.

**Conclusions:**

This study shows that while PubMed returns the most robust list of publications, variations in the format of reported funding support and indexing practices meant no one resource was sufficient to capture all publications that cited a given NIH project grant number. Librarians looking to help build grant-specific publication lists will need to use multiple resources and be aware of the most frequently reported grant variations to identify a comprehensive list of supported publications.

## INTRODUCTION

Funding awards from the US National Institutes of Health (NIH) typically require publications and other outcomes of the grant to be regularly reported to remain in good standing throughout the award period. Similarly, for renewal considerations, a comprehensive list of publications produced under the award are often needed to demonstrate successful outcomes and the impact of funded research. To support these requirements, large multidisciplinary research centers may turn to their affiliated libraries for assistance in identifying publications produced under their NIH program projects or center grants. Such program grants (also known as P-series) are large, multi-project efforts that may support several hundred researchers with various community and institutional affiliations. Searching in the public record remains an accepted and reliable way to identify publications that researchers produce under such funding awards. However, searchers need a clear understanding of how NIH awards of interest are indexed in various data sources to build effective search strategies.

Since first offering funding acknowledgment indexing in 2008, Web of Science remains the most widely recognized and recommended source for analyzing publication funding acknowledgments [1–10]. However, only one study reviewed to date searched for acknowledgments to a specific grant number rather than to the larger funding organization [1]. In either case, whether searching by specific grant number or funding organization name, the acknowledgment field remains unstandardized, meaning that the keywords necessary for searching this field are subject to a great deal of variations, including those in grant number formatting and punctuation [2]. Studies examining funding acknowledgments to a specific organization found the number of name variations could range from several hundred to several thousand [2, 3, 8]. Further reports indicate indexing errors in 12%–32% of publication acknowledgments, largely due to author inconsistencies regarding the funder-requested formats [1, 4, 5, 11].

Comparisons of acknowledgment indexing between Web of Science and PubMed highlight the language, country of origin, document type, and disciplinary biases found in both databases [4, 7–9, 12]. However, as NIH programs and grants primarily support English-language journal publications, either database remains a viable source for creating comprehensive publication lists from these funding awards. For NIH awards, the NIH Research Portfolio Online Reporting Tool for Expenditures and Results (NIH RePORTER) provides an additional means of identifying supported publications.

This study describes an attempt to construct a comprehensive list of publications produced by research members under an NIH program award to the Center for AIDS Research (CFAR) at Emory University. This center reached out to the Woodruff Health Sciences Center Library for assistance with preparing the necessary publications list for an upcoming grant renewal. After initially pulling publications from NIH RePORTER, it was noticed that many grant-citing publications were overlooked. Additional publications were, therefore, sought from both Web of Science and PubMed. The publication lists provided by these three data sources were compared to improve understanding of current database indexing practices and help others build appropriate search strategies for identifying specific funding award–associated publications. The goals of this study were (1) to identify the level of coverage provided by each data source and (2) to design appropriate search methods that could be adapted for future inquires of NIH grant-supported publications.

## METHODS

### Searching in NIH RePORTER

NIH RePORTER provides lists of publications produced as a result of NIH support. The NIH RePORTER public query interface was used to search for all results under grant number P30AI050409 since 2008, and all results under the “publications” tab were exported for review. As of the collection date of June 2018, a total of 860 publications were listed as being published between 2008 and 2017. PubMed identification numbers (PMIDs) for these publications were exported.

### Grant number variations

**Figure 1:**
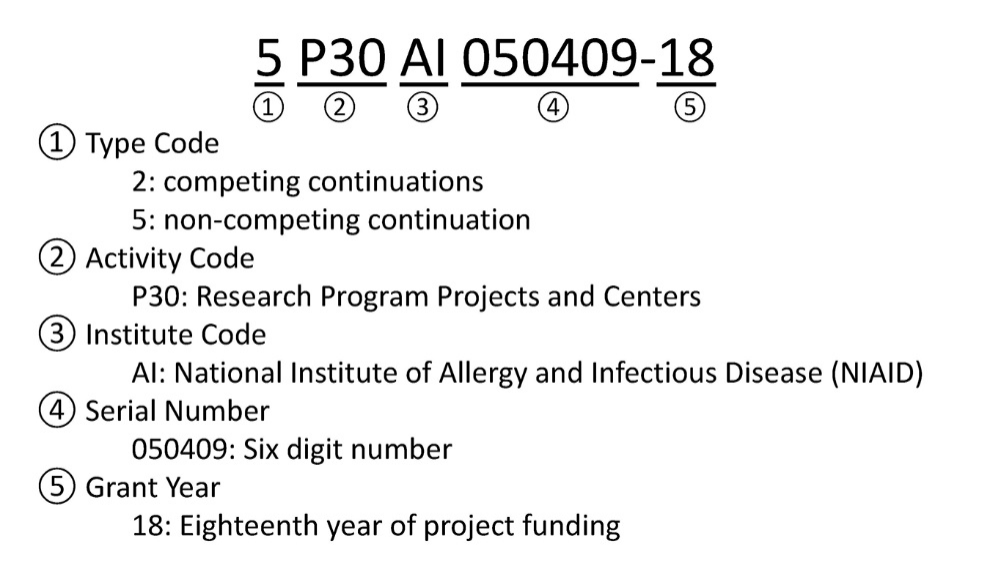
Components of a National Institutes of Health (NIH) grant number

Publication lists in NIH RePORTER are mapped to a single standardized identifier, or grant number, issued by NIH. By contrast, funding acknowledgments in Web of Science and PubMed are based in part on free-text indexing, which is subject to grant number variations. To illustrate, Figure 1 presents the common structure of all NIH grants, exemplified by the CFAR grant of interest. The Activity Code, Institute Code, and Serial Number are used as the official number in NIH RePORTER (i.e., P30AI050409) as these elements are constant throughout the lifespan of the award, whereas the Type Code and Grant Year elements can vary from year to year. Annual variations, subsets, or errors in any of these elements result in variations in the indexing databases that must be accounted for in the search string.

### Searching in Web of Science Core Collection

Initial identification of Web of Science search terms was performed using the NIH RePORTER dataset. A Web of Science results list was created using an advanced search query by the PMIDs provided by NIH RePORTER. The “Analyze Results” feature was then used to collect and record the relevant variations in funding acknowledgment. This feature is available under the “Analyze Results” link at the top of all Web of Science results lists: users can select the “Grant Numbers” filter to view and export all indexed grant numbers associated with the search results. All P30AI050409 variations were recorded. These recorded variations were then tested in a new search string to identify additional indexed publications that acknowledged the grant of interest but were not listed in NIH RePORTER. Variations discovered in PubMed were also tested and added as appropriate.

Further variations, given observed patterns, were also tested (e.g., alphanumeric substitutions, missing zeros). Wildcard (*) searching was attempted but cannot be recommended, as only right-hand truncation (e.g., P30AI*) is currently supported in the grant number search field and generally returned results unrelated to the award of interest. All required keywords for a comprehensive publication list were recorded, revealing 17 variations to the CFAR grant number in 787 publications. All publications returned by these keywords were found to correctly relate to the award of interest. The final Web of Science search strategy was:

fg=(50409 OR 050409 OR AI50409 OR A150409 OR AI050409 OR A1050409 OR AI0050409 OR A01050409 OR P30AI50409 OR P30AI050409 OR P30A1050409 OR P30AL050409 OR 2P30AI050409 OR 2P30A1050409 OR 5P30AI50409 OR 5P30AI050409 OR 5P30A1050409)

### Searching in PubMed

PubMed advanced search options provide a list of unique grant numbers. By entering leading characters into the “Grant Number” field and selecting “Show Index List,” users can browse possible complete number variations. From this index, variations to the P30AI050409 grant number were tested and added to a saved search string, and publication results were examined. Attempts were made to discover all listings including the numeric string “50409,” and results were reviewed for matches to the award of interest. This was done through manual exploration of the index list, as left-hand truncation, internal truncation, and wildcard searching (*) are not currently supported by PubMed. Right-hand truncation (e.g., “P30AI*” or “50409*”) returned results that were not related to the grant of interest or were more accurately captured through more specific keywords (e.g., “50409 04a1”).

Variations discovered in the Web of Science database were also tested and added as appropriate. Hypothetical variations, given observed patterns, were also tested (e.g., alphanumeric substitutions, missing 0s). In total, 46 variations in the CFAR grant number were discovered, returning 920 publications from 2008 to 2017. Results returned by each variation were recorded, reviewed, and compared; this process revealed that 15 unique keywords were needed to return a comprehensive publication list. For example, while ai050409-04a1 (denoting the 4th year of active funding) and ai050409-07 (denoting the 7th year of active funding) were listed separately in the advanced search index, a search for “ai050409” returned both, meaning that these 2 variations required only a single keyword. All 920 publications returned by these keywords were found to correctly relate to the award of interest. The final PubMed search strategy was:

((((((((((((((“50409 04a1”[Grant Number]) OR “50409 10”[Grant Number]) OR ai50409[Grant Number]) OR ai050409[Grant Number]) OR ai0050409[Grant Number]) OR aL050409[Grant Number]) OR a150409[Grant Number]) OR a1050409[Grant Number]) OR a01050409[Grant Number]) OR p30ai50409[Grant Number]) OR p30a1050409[Grant Number]) OR p30AL050409[Grant Number]) OR “2p30ai 050409”[Grant Number]) OR 2p30a1050409[Grant Number]) OR 5p30ai50409[Grant Number])

## RESULTS

A total of 986 unique publications acknowledging the award of interest were identified from PubMed, Web of Science, and NIH RePORTER. Table 1 shows a breakdown of results by each database.

**Table 1:**
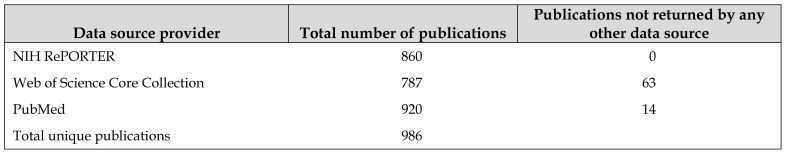
Publication results by data source

Data source provider	Total number of publications	Publications not returned by any other data source
NIH RePORTER	860	0
Web of Science Core Collection	787	63
PubMed	920	14
Total unique publications	986	

**Figure 2:**
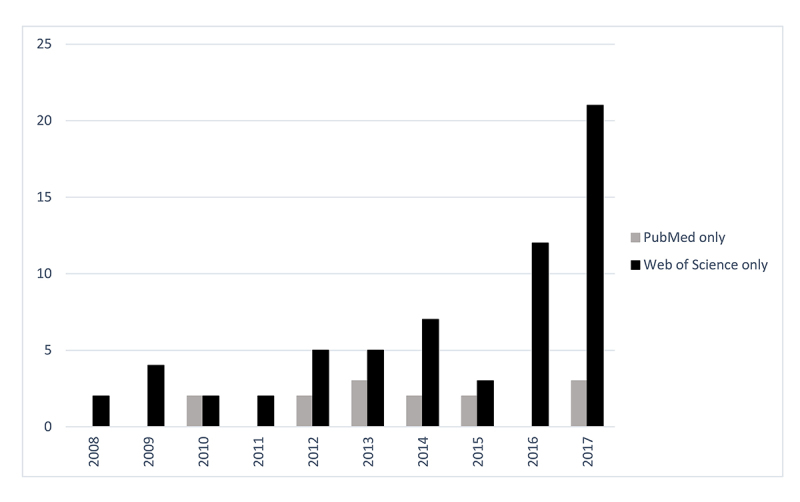
Unique publications by data source per year Web of Science returned unique results for all years, whereas PubMed returned unique results for only some years.

While PubMed returned the largest number of publications, Web of Science returned the most publications that were not identified by the other 2 data sources. Although this observation held true across all 10 years, Web of Science offered a noticeably larger proportion of unique results in more recent years (Figure 2). Of these uniquely returned publications (n=63), over half (n=40) were indexed in MEDLINE but lack associations to the award of interest despite this information being available in the full text. Two publications were expected to be indexed in MEDLINE but were listed as “In-Process.” The remaining publications were listed as “PubMed-Not-MEDLINE” (n=7) or did not appear in PubMed at all (n=14). NIH RePORTER listed only 3 publications that were not identified by PubMed; all 3 were returned by Web of Science. Each of these 3 publications were indexed in MEDLINE, but either no associated funding information was indexed (n=2) or the award of interest was not among those listed (n=1). In all 3 cases, the CFAR award acknowledgment was available in the publication full text. Thus, none of the 3 data sources singularly provided a full list of acknowledging publications when searched by the CFAR funding number.

Variations in funding acknowledgments stemmed primarily from truncations of the official NIH grant number (Table 2). The most common variation was a truncated version using only the NIH Institute Code and Serial Number (AI050409). The most common errors were the letter “I” in the Institute Code being substituted with the number “1” and the omission of the leading 0 in the 6-digit Serial Number. In total, 21 variations to the CFAR grant number were needed to build a complete data set between the 2 databases.

**Table 2:**
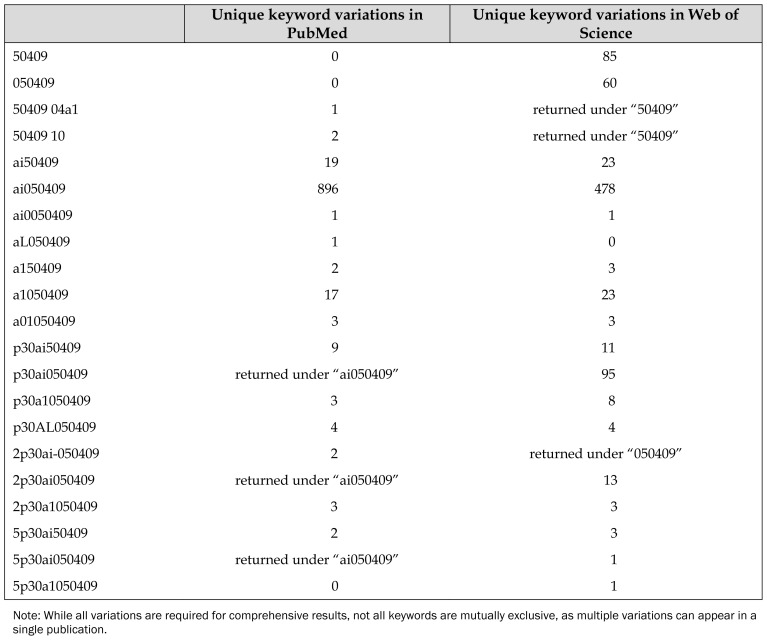
Unique keyword variations by data source

	Unique keyword variations in PubMed	Unique keyword variations in Web of Science
50409	0	85
050409	0	60
50409 04a1	1	returned under “50409”
50409 10	2	returned under “50409”
ai50409	19	23
ai050409	896	478
ai0050409	1	1
aL050409	1	0
a150409	2	3
a1050409	17	23
a01050409	3	3
p30ai50409	9	11
p30ai050409	returned under “ai050409”	95
p30a1050409	3	8
p30AL050409	4	4
2p30ai-050409	2	returned under “050409”
2p30ai050409	returned under “ai050409”	13
2p30a1050409	3	3
5p30ai50409	2	3
5p30ai050409	returned under “ai050409”	1
5p30a1050409	0	1

Note: While all variations are required for comprehensive results, not all keywords are mutually exclusive, as multiple variations can appear in a single publication.

## DISCUSSION

Of the 3 investigated data sources offering lists of publications supported by the CFAR award of interest, none were sufficient to identify all publications supported by the award. PubMed returned the greatest number of results (93% of the total identified), whereas WoS returned an additional 6% that were not listed elsewhere. The NIH RePORTER listings captured only 87% of identified publications that acknowledged support from the CFAR award. The presence of unique results in other databases, regardless of publication age, suggests that this discrepancy was likely due to different indexing practices, rather than different data source processing times. Each data source identifies and indexes funding acknowledgments using different methodologies: NIH RePORTER relies on several extant databases such as eRA databases, MEDLINE, PubMed Central (PMC), the NIH Intramural Database, and iEdison [13]; PubMed uses a combination of in-text harvesting and information derived from PMC and My NCBI [14]; and Web of Science uses in-text harvesting supplemented by funding data available from MEDLINE and Researchfish [15]. Both PubMed and Web of Science rely heavily on the harvesting of in-text acknowledgments, which are subject to reporting errors as evidenced by the large number of variations found in both data sources.

Perhaps most surprising was that the publications that NIH RePORTER overlooked appeared in MEDLINE and PMC, despite both data sources being described in NIH RePORTER documentation. Variations in grant number format, while an obvious source of difficulty, could not, therefore, fully account for the discrepancy between databases. Of the publications that NIH RePORTER captured, 95% listed the variation AI050409, yet 48% of those indexed in PubMed but overlooked by NIH RePORTER included this same variation. Even when the funding acknowledgment is correctly listed in the full text or correctly appears in the PubMed record, the acknowledgment might still be overlooked by other databases. Considering these observations, it is recommended that multiple data sources be used when attempting to construct a comprehensive bibliography of publications that result from funding support.

Despite the indexing discrepancies of all three data sources, the biggest difficulty in identifying publications produced by a particular award is author-supplied variations in funding acknowledgments. Thus, the greatest challenge to searching multiple data sources is creating effective search queries for each source. The findings presented here highlight some of the most common truncations and errors that are made when acknowledging NIH funding support, including expanded or truncated grant numbers, missing or extra zeros, and alphanumeric substitutions.

These observations could be applied to inform likely keywords for identifying publications that acknowledge other NIH awards of interest. However, given the unpredictability of human error and in the absence of standardization in indexing acknowledgment fields, initial manual testing for likely variations of each specific grant number is recommended. At the very least, searchers will need to execute an iterative process of searching, identifying common errors, and expanding on likely substitutions to ensure the most complete list of publications. Furthermore, when searching by other grant numbers, particularly those using non-NIH structures, these recommendations may need to be modified to account for false positives (i.e., publications matching grant number elements but not related to the award of interest).

Finally, it is important to recognize that the formal reporting of funding support is still subject to disciplinary differences, organizational cultures, and individual author diligence. Thus, even the most robust search strategies might not identify all publication outputs from a given award. Studies as recent as 2015 report that only about 50% of natural sciences publications include formal support acknowledgments [5, 12]. Searching by grant number may, therefore, uncover only a portion of the supported output and cannot fully replace investigator progress reports and other reporting. However, with documented funding support being a growing area of study, future investigations are needed to compare indexing strategies of Scopus (Elsevier), Dimensions (Digital Science), and Google Scholar to further understand the landscape of funding acknowledgments. It is expected that these sources would provide additional unique results but will also require carefully identifying and testing keyword variations.
